# Flexibility in Animal Signals Facilitates Adaptation to Rapidly Changing Environments

**DOI:** 10.1371/journal.pone.0025413

**Published:** 2011-09-28

**Authors:** Darren S. Proppe, Christopher B. Sturdy, Colleen Cassady St. Clair

**Affiliations:** 1 Department of Biological Sciences, University of Alberta, Edmonton, Alberta, Canada; 2 Department of Psychology, University of Alberta, Edmonton, Alberta, Canada; 3 Department of Biological Sciences, University of Alberta, Edmonton, Alberta, Canada; University of Regina, Canada

## Abstract

Charles Darwin posited that secondary sexual characteristics result from competition to attract mates. In male songbirds, specialized vocalizations represent secondary sexual characteristics of particular importance because females prefer songs at specific frequencies, amplitudes, and duration. For birds living in human-dominated landscapes, historic selection for song characteristics that convey fitness may compete with novel selective pressures from anthropogenic noise. Here we show that black-capped chickadees (*Poecile atricapillus*) use shorter, higher-frequency songs when traffic noise is high, and longer, lower-frequency songs when noise abates. We suggest that chickadees balance opposing selective pressures by use low-frequency songs to preserve vocal characteristics of dominance that repel competitors and attract females, and high frequency songs to increase song transmission when their environment is noisy. The remarkable vocal flexibility exhibited by chickadees may be one reason that they thrive in urban environments, and such flexibility may also support subsequent genetic adaptation to an increasingly urbanized world.

## Introduction

Darwin attributed the presence of exaggerated visual and acoustic traits in males of many animals to sexual selection, which he distinguished from natural selection because of the apparent irrelevance, and even detriment, of those traits for survival [1]. Subsequent scholars have shown that many sexually-selected traits are honest signals of individual quality, and are effective targets for assessment by females [2]. In Darwin's most famous example, the peacock, the number of spots in the tail is correlated with the survival of offspring produced by that male [3]. Because sexually-selected traits also influence survival, stabilizing selection may limit the ‘runaway’ process that would otherwise apply to sexually-selected traits [4]. Such stabilizing selection appears to apply to bird song, for which females prefer population-specific ideals of note composition, frequency and duration [5].

Despite the strong historic selection for particular song characteristics, birds living in more urbanized landscapes now appear to be under selection to overcome the effects of anthropogenic noise. The low-frequency noise caused by vehicle traffic is a particular problem, and bird species that persist adjacent to roads appear to produce higher-frequency songs than species that live in the surrounding landscape [6]. In some species, individuals in noisy environments use higher-frequency song [7], presumably to reduce the masking effects of traffic noise (for masking effects of noise see Lohr et al. [8]). Recent experimental work has shown that frequency shifting can be achieved via short-term behavioural plasticity within individuals [9], [10], suggesting that birds now face a selective trade off. Whereas high frequency songs reduce the masking of traffic noise, they also attenuate more rapidly, reducing the range over which they can be heard [11]. And perhaps more importantly, these higher frequency songs differ from population-specific archetypes that signal dominance, important for deterring rivals and attracting females. One potential mechanism to balance sexual selection with masking anthropogenic noise is to switch plastically between ‘modified’ and ‘normal’ signals as environmental conditions change.

We addressed the possibility of a plastic response to dual selective pressures in the black-capped chickadee (*Poecile atricapillus*), a forest-dependent species that has successfully adapted to urban habitats throughout North America [12]. We have previously demonstrated that chickadees in noisier areas sing at higher frequencies [13]. However, the two documented markers of dominance in the chickadee's *fee-bee* song (pitch ratio and amplitude ratio between the *fee* and the *bee* notes; [14], [15]) appear to be more difficult to maintain at higher frequencies [16]. Thus, we hypothesize that chickadees adjust signal characteristics to their ambient environment to maintain sexually selected markers of dominance while avoiding overlap with anthropogenic noise. In particular, we predict that birds would sing their *fee-bee* songs at higher frequencies under noisy conditions and at lower frequencies when it is quieter.

A recent study found that song duration was shorter in great tits (*Parus major*) inhabiting noisy, urban locations than in quiet, rural locations [17]. Because urban habitat tends to be more open than rural habitats, the authors speculated that adaptation to habitat likely explained differences in song duration. Sites in the current study differed in noise levels over time, but they did not differ in habitat structure, which provided an opportunity to test whether birds sing songs differ in durations as a function of noise alone. We hypothesized that songs would be shorter in noisier sites because they would be more detectable and incur less energetic cost when placed in noise gaps between bouts of traffic.

## Methods

To evaluate these hypotheses in a natural situation, we recorded black-capped chickadee *fee-bee* songs at 22 roadside locations (<100 m from a high use road, >20,000 vehicles/day, 2007 City of Edmonton Traffic Flow Map) where a male black-capped chickadee was heard singing loudly on multiple visits, and presumed to be a territorial resident. Recording sessions were conducted from April 23–May 22, 2009, in Edmonton, Alberta, Canada, from 0400–0800 hours, a timeframe that correlates with an increase in the volume of low-frequency masking noise produced by higher levels of traffic volume. Because several environmental variables (e.g., temperature, light) change over the same timeframe, we recorded each location on a weekend and a weekday (hereafter day type) when only traffic patterns were presumed to differ. Male *fee-bee* songs were recorded via an automated recording unit attached to a tree at 3–4 m above the ground (44,100 Hz sampling rate; 16 bit sampling depth; frequency range: 20–20000 Hz, omni-directional microphone; Song Meter SM1; Wildlife Acoustics, Massachusetts, USA).

Eighteen to 20 songs were analysed for each day type from each site. To ensure that songs came from the full range of ambient noise that occurred at each site and to minimize the use of repeated songs from any one particular bout, songs were selected equally from each hour of recorded time. By spreading selected songs over time and multiple bouts, we reduced the possibility that our analysis was significantly affected by social factors, such as song matching between neighbouring males. To minimize frequency differences due to temporal factors alone, selected songs from the two recorded mornings at each site were paired by randomly selecting a song for one day type (e.g., weekend) and then selecting the song closest in time from the other day type. Since within-song frequency measures are highly correlated [18], we measured only *bee* note peak frequency (frequency at the loudest amplitude; power spectrum: 32,768 points, frequency resolution 1.3 Hz, high pass 2,200 Hz [19]) and duration of the entire song (spectrograph: 1,024 points, minimum cutoff = −50 dB, visible frequency range 2,000–5,000 Hz).

To determine the temporal scale over which birds responded to traffic noise, we recorded ambient noise over two time scales; one minute prior to song production (instantaneous) and for the quarter hour overlapping song production (average). Similar to Slabbekoorn [20], we measured ambient noise levels in 1 kHz bandwidths (fast Fourier transform; 65,536 points, frequency resolution of 0.7 Hz) in SIGNAL 5.0 (Engineering Design 2008, Berkeley, California) to obtain the mean noise level in (1) six random, one-second samples that were free of *fee-bee* vocalizations within the minute prior to each sampled song (instantaneous; n = 851), and (2) the quarter hour containing the song (average; n = 851). The 1–2 kHz bandwidth in *fee-bee* free recordings was highly correlated with 2–3 (Pearson's *r* = 0.929) and 3–4 kHz (Pearson's *r* = 0.900) bandwidths, and thus, was used as a proxy to estimate noise levels in the range of chickadee vocalizations for both instantaneous and average noise. All measurements were converted from root mean square to decibels (dB) via the formula dB = −20 log10 (Volts) and standardized (i.e., lowest dB set to 0).

For analysis, site was the unit of replication, with 36–40 song replicates within each site allowing us to examine the overall pattern of change in relation to noise or day type. To account for the non-independence of within site song measurements, we employed a general linear mixed model (GLMM) with site included as a random effect (STATA 10.1: xtmixed [21]). GLMM results determined the effects of day type, noise, and a noise×day type interaction on *bee* note peak frequency and song duration. Time was also included in the model as a covariate to account for variance due to temporal patterns. Our measures of instantaneous and average noise were highly correlated (Pearson's *r* = 0.876) and thus, for each model we retained only the variable with the lower Akaike's Information Criterion (AIC) value obtained from a univariate regression [22].

## Results

**Figure 1:**
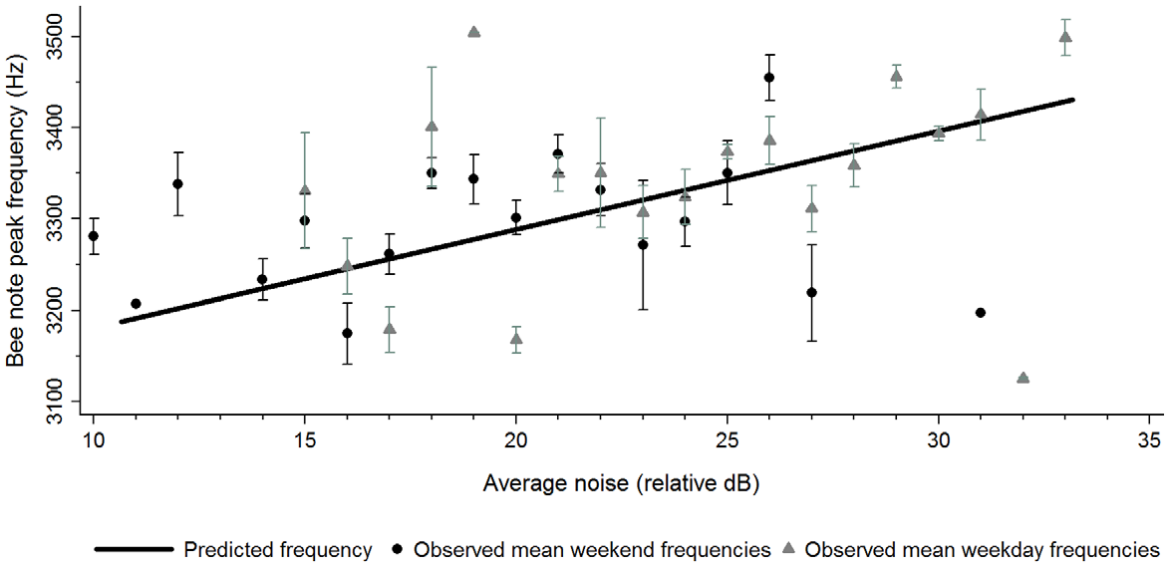
Relationship between *bee* note peak frequency and average ambient noise levels. The line predicting *bee* note peak frequency is derived from a general linear mixed model and data points correspond to the observed mean *bee* note peak frequency at each decibel (±1 SE) averaged for all songs in all sites on weekend (black circles) and weekday (grey triangles) recordings.

For *bee* note peak frequency, average noise was a better fit than instantaneous noise (ΔAIC = 48.58), suggesting that song frequency was more strongly related to general ambient noise conditions (15 min) than the noise level immediately preceding song production. A general linear mixed model (GLMM) revealed that *bee* note peak frequency increased by 13.81 Hz/decibel as average noise levels increased (*z* = 2.01, *P* = 0.04; Figure 1), but did not vary significantly with any other variable (*z*<1.10, *P*>0.27).

**Figure 2:**
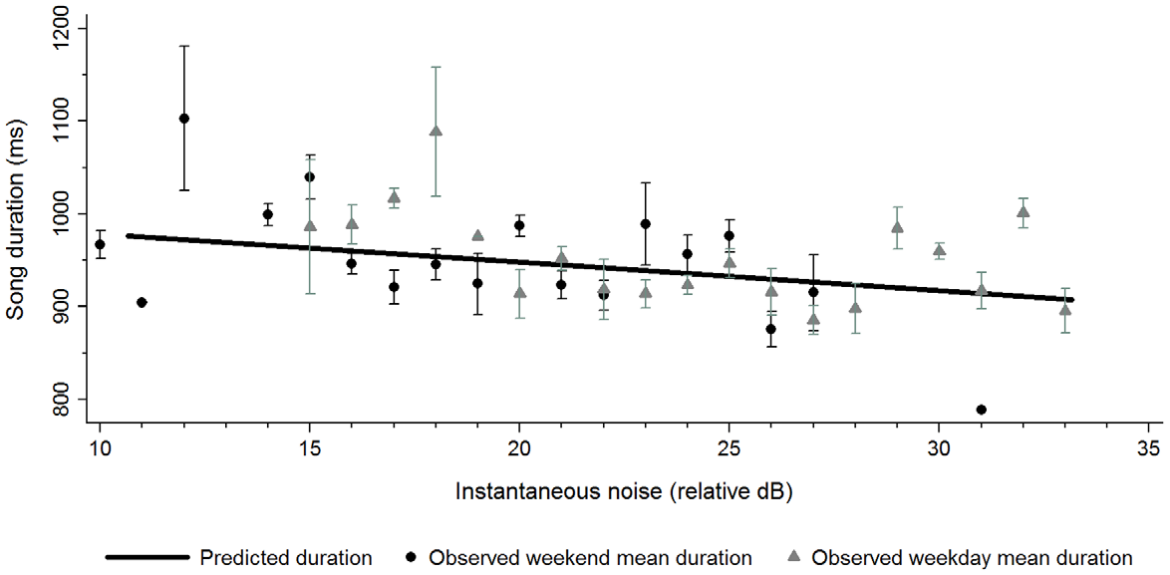
Relationship between song duration and instantaneous noise. The line predicting song duration is derived from a general linear mixed model and data points correspond to the observed mean song duration at each decibel (±1 SE) averaged for all songs in all sites on weekend (black circles) and weekday (grey triangles) recordings.

For song duration, instantaneous noise provided a better fit than average noise (ΔAIC = 7.63), suggesting that song duration was modified more quickly than frequency in response to ambient noise. Song duration decreased by 3.09 ms/decibel as instantaneous noise levels increased (z = 1.93, P = 0.05; Figure 2). This result falls on the line of significance under the statistical criteria of α = 0.05. Song duration did not vary significantly with any other variable (*z*<1.06, *P*>0.29). Noise did not interact with day type for peak frequency (*z* = 0.84, *P* = 0.40) or duration (*z* = 0.25, *P* = 0.81), indicating that other environmental factors that change within a morning cannot explain the observed relationships.

## Discussion

Our results reveal that black-capped chickadees reduced song duration over the timescale of seconds and increased song frequency over the course of minutes to accommodate increases in ambient noise; but sang lower-frequency, longer songs when noise abated. When noise levels are low, the latter songs presumably remain detectable over longer distances and more easily maintain dominance markers [15] that enhance both territorial defense and female attraction.

Chickadees responded to average noise levels with changes in *bee* note frequency, but responded more quickly to changes in noise conditions for song duration. The longer time frame associated with frequency may stem from the highly conserved frequency ratio of the *fee* and *bee* notes [23]. Ambient noise near a roadway can change over the duration of a single *fee-bee* song, but birds that alter frequency ratios between the *fee* and the *bee* notes may be less attractive to females [14], [15]. This stereotypy may limit the adjustments that can be made mid-song. In this study, chickadees averaged 2.26±0.26 (SE) songs/minute from 0500–0800 hours. This rate of song repetition may make it possible for chickadees to respond to traffic noise on an intermediate time scale between one (instantaneous) and fifteen (general) minutes. By matching their song frequencies to average noise over a period of at least several minutes, birds may achieve optimal song frequencies without adjusting note ratios.

Song characteristics that convey dominance may be easier to maintain at lower frequencies, but maintaining the ability to vocalize across a wide range of frequencies is also important for establishing dominance. Black-capped chickadees use a wide range of frequencies even when anthropogenic noise levels are low [24]. Since multiple males inhabit adjacent territories, song bouts between rival males are commonplace. Horn et al. [25] found that male chickadees accurately matched the frequencies of played back song from conspecific males. Further research has shown that dominant males are more likely to match the songs of rival males than subordinates [26], and that females assess matching bouts to evaluate male quality [27]. In a recent study, we found that chickadees sang *bee* notes at lower overall frequencies in quieter locations near Edmonton, Alberta, Canada. However, vocalizations still ranged from 2823–3454 Hz in these quiet sites, with over 50% of songs being sung above 3208 Hz [13]. The range in the current study was 2839–3715 Hz, with 50% of songs sung above 3344 Hz. In both studies, chickadees employed a wide range of vocal frequencies. The ability to sing across many frequencies appears to be ubiquitous among black-capped chickadees, allowing individuals to signal dominance by matching conspecific male songs.

In areas with elevated anthropogenic noise, higher frequency songs become more advantageous. They are easier to detect due to spectral separation from lower frequency sounds produced by anthropogenic noise [8]. A male that can be heard possesses an immediate advantage over one that cannot. Singing at higher frequencies in noisy areas maximizes the likelihood that neighboring males will be aware of the singer's presence. Perhaps males in noisy areas use higher frequency songs to gain the attention of females, but retain the use of lower frequency songs to more easily convey dominance when noise levels are low. In sum, chickadees appear to benefit from possessing a wide range of song frequencies in both noisy and quiet locations.

Song duration is not as stereotyped as frequency ratios [16], potentially allowing for easier adjustment after song initiation. Some authors suggest that producing song is only mildly costly for birds [28], but the energetic costs that are incurred may be closely related to song duration [29]. Chickadees that shorten their songs immediately in response to heightened traffic noise might save energy over an entire breeding season, particularly when they inhabit higher latitudes where cold temperatures exert higher oxygen demands [30]. Alternatively, shorter songs may more easily fit into gaps between noise events (i.e., passing vehicles), a strategy that is consistent with the shortening of songs chickadees exhibit when overlapped by conspecifics [31]. In another vocal species, the duetting grasshopper (*Chorthippus biguttulus*), the ability to detect onset and offset of male vocal signals is important for female response [32]. To our knowledge, no work has previously addressed this potential adaptation in the context of road noise.

The remarkable degree of vocal flexibility exhibited by chickadees undoubtedly contributes to their abundance in urban areas [12], but it may also support subsequent adaptation to a more urbanized world. As ambient noise increases, individuals with the capacity to produce the highest-frequency songs may experience selective advantages that augment behavioural flexibility. Some evidence for such directional selection is provided by a recent study of male great tits (*Parus major*), which responded most aggressively to the playback frequency that correlated with the level of anthropogenic noise in their immediate environments [33]. Because females of many songbird species assess male quality by eavesdropping on these dominance interactions [27], selection induced by anthropogenic noise and female choice may converge.

Future work could profitably address variability in vocal flexibility among males that is attributable to behavior, development, and evolution; and evaluate comparable differences among female preferences. Comparative work might assess differences in vocal flexibility among species of different conservation status and in relation to different combinations of acoustic and spatial habitat degradation. More information of this sort will help to mitigate a variety of anthropogenic effects on birds and may also reveal unexpected adaptive capabilities of wildlife to anthropogenic impacts.
